# Animal-Free Skin Sensitization Testing: In Chemico and In Silico Integrated Approach

**DOI:** 10.3390/toxics14070585

**Published:** 2026-07-02

**Authors:** Gabriella Lisboa dos Santos, Gabriela de Oliveira Prado Corrêa, Franciane de Oliveira Cortez, Tugstênio Souza, Bruna Bosquetti, João Antonio Dassie Felippi, Gabriela Trindade de Souza e Silva, Pamela Ferreira do Nascimento, Marcio Adriano Andréo, Marcelo Dutra Duque, Carolina Motter Catarino, Andrezza di Pietro Micali Canavez, Patricia Santos Lopes

**Affiliations:** 1 Department of Pharmaceutical Sciences, Federal University of São Paulo, Diadema Campus, Diadema 09913-030, Brazil; gabriella.99lisboa@outlook.com (G.L.d.S.);; 2 Pre-Clinical & Product Safety-Grupo Boticário, R. Alfredo Pinto, 2258-Parque da Fonte, São José dos Pinhais 83050-320, Brazil; gabriela.correa@grupoboticario.com.br (G.d.O.P.C.);; 3 Faculdade de Ciências Farmacêuticas, State University of Campinas, Rua Cândido Portinari, 200, Cidade Universitária Zeferino Vaz, Campinas 13083-871, Brazil; 4 Programa de Pós-Graduação em Medicina Translacional, Departamento de Medicina, Escola Paulista de Medicina, Universidade Federal de São Paulo, São Paulo 04021-001, Brazil

**Keywords:** skin barrier, skin sensitization, *Baccharis trimera*, in chemico, in silico, DPRA, safety testing

## Abstract

The increasing prohibition of animal testing for cosmetic products has driven the development of alternative approaches to ensure consumer safety. Skin sensitization is one of the most critical toxicological endpoints to evaluate, requiring rigorous assessment to ensure the safety of cosmetic ingredients. This study proposes an Integrated Testing Strategy (ITS) that combines in chemico (Direct Peptide Reactivity Assay—DPRA) and in silico approaches (a six-platform computational panel) to evaluate the sensitization potential of substances. Initially, the in chemico methodology was validated through a partial proficiency demonstration to ensure experimental reliability. Subsequently, this ITS was applied to the *Baccharis trimera* extract and its major marker, 3-Caffeoylquinic acid (chlorogenic acid/3-CQA). Our results demonstrate that the DPRA alone is insufficient to classify the sensitization potential of complex mixtures, as recommended by OECD guidelines. The integration of in silico data proved essential to interpret the reactivity of the botanical matrix, revealing that the sensitization potential observed in the extract does not stem solely from 3-CQA, but likely results from the synergistic contribution of more lipophilic caffeoylquinic acid isomers. This approach demonstrates that integrating experimental and computational methods is fundamental for a robust safety assessment, offering an efficient, animal-free strategy for the early screening of cosmetic ingredients and for refining the interpretation of toxicological data in complex chemical environments.

## 1. Introduction

Skin sensitization is a toxicological endpoint triggered by exposure to exogenous substances and is associated with a range of clinical signs and symptoms collectively known as allergic contact dermatitis. Several cosmetic ingredients can induce such reactions, including p-phenylenediamine, which is widely used as a dye, particularly in hair coloring products [1]. Therefore, among the evaluations to which cosmetic products and ingredients are subjected prior to commercialization, allergenic potential represents one of the most important safety parameters.

In contrast to common local irritation, which results from direct cellular damage, allergic contact dermatitis arises from an adaptive immune response and involves two distinct phases: (i) induction, in which initial exposure to an allergen triggers an innate immune response and leads to the formation of memory T cells, and (ii) elicitation, in which subsequent exposure provokes the clinically observable effects [2]. Traditionally, elicitation induced by a substance has been evaluated in mice using the Local Lymph Node Assay (LLNA) [3]. However, following the elucidation of the skin sensitization Adverse Outcome Pathway (AOP) in 2012, not only have non-animal methods been developed, but the overall assessment strategy has also shifted toward the perspective of New Approach Methodologies (NAMs). Consequently, rather than proposing a single test or method, this approach emphasizes the integration of existing information with newly generated data obtained from a battery of assays that individually evaluate each key event of the endpoint [4].

The AOP describes four biochemical stages, or key events, that lead to T-cell activation and, consequently, to the sensitization process: (1) covalent binding of the substance to nucleophilic proteins after penetration through the stratum corneum, resulting in hapten formation; (2) activation of keratinocytes through the Keap1/Nrf2 signaling pathway, triggering the antioxidant/electrophile response element (ARE) and the expression of inflammatory mediators; (3) recognition of the antigen by skin dendritic cells, particularly Langerhans cells, leading to the production and differential expression of surface markers; and (4) migration of dendritic cells to the lymph node, antigen presentation, and T-cell proliferation, ultimately resulting in the elicitation of the allergic response upon subsequent exposure to the substance [4].

The elucidation of these stages has enabled the development and validation of non-animal assays targeting the first three key events. OECD Test Guideline 442C [5] describes the Direct Peptide Reactivity Assay (DPRA), an in chemico method that employs cysteine and lysine-containing peptides to mimic nucleophilic skin proteins. Sensitizing compounds react by forming covalent bonds with the peptides (the first AOP event), leading to their depletion from the reaction medium, which can be quantified by high-performance liquid chromatography (HPLC). According to the guideline, to ensure reliable application of this technique, each laboratory must demonstrate proficiency by following the prediction model specified in the guideline to validate the generated results. However, this model is considered applicable only to single-constituent substances or mixtures with known and stable compositions, owing to the required molar ratio between the test substance and the peptide. Consequently, multicomponent systems, such as plant extracts, must be thoroughly characterized to enable appropriate testing [5].

A promising alternative aligned with the 3Rs principle is the in silico approach, which leverages predictive software and chemical databases to categorize molecules based on structural features linked to sensitization potential. Quantitative Structure–Activity Relationship [(Q)SAR] models form the core of this methodology, predicting the biological behavior of compounds by analyzing structural similarities to known toxicological data [6,7]. In this study, we employed an integrated in silico screening strategy [8] utilizing a battery of specialized computational platforms, including ADMET Predictor™ [9,10], OECD QSAR Toolbox [11], StopTox [12], VEGA QSAR [13,14], Pred-Skin [15,16], and the Danish QSAR [17] database in addition to in chemico test DPRA. This multi-tool framework is particularly relevant for the cosmetic industry, which faces a rising demand for ‘green’ ingredients derived from biodiversity [18]. These raw materials are typically complex mixtures containing lipids, carbohydrates, phenolics, and terpenoids, often lacking comprehensive experimental toxicological data [19]. Among these, phenolic compounds are especially prioritized for their antioxidant capacity and free-radical scavenging properties, making them highly sought-after for anti-aging cosmetic applications [20].

*Baccharis trimera* (Less.) DC., a species native to Brazil, has developed specialized secondary metabolic pathways as an evolutionary response to environmental pressures. Due to Brazil’s geographic positioning across the equator and within tropical zones, the native flora is subjected to persistent, high-intensity solar and ultraviolet (UV) radiation throughout the year. To mitigate the resulting oxidative stress, *B. trimera* synthesizes a rich profile of polyphenols, with chlorogenic acid and its caffeoylquinic derivatives acting as natural photoprotective filters and free radical scavengers [21,22]. Consequently, exploring these specialized extracts provides a robust, scientifically grounded rationale for their incorporation into topical cosmetic formulations aimed at sun protection and anti-aging, specifically tailored for populations exposed to elevated solar incidence.

To effectively replace animal-based models, both the OECD and several international organizations recommend the integrated use of in chemico, in vitro, and in silico techniques, known as Integrated Approaches to Testing and Assessment (IATA) [23]. Joint interpretation of the resulting data enables accurate prediction of the skin sensitization potential of cosmetic ingredients. In addition, there is a need for comprehensive evaluation of available safety assessment methods to adapt them for application to complex mixtures and natural products.

Accordingly, this study aimed to demonstrate partial DPRA proficiency by using a subset of the reference substances specified in the protocol and to integrate these experimental data with a comprehensive in silico panel—comprising ADMET Predictor™, OECD QSAR Toolbox, StopTox, Pred-Skin, Vega QSAR, and Danish QSAR. Furthermore, the applicability of this Integrated Testing Strategy (ITS) was evaluated for predicting the skin sensitization potential of the hydroalcoholic extract of *B. trimera* and its major constituent, 3-Caffeoylquinic acid (chlorogenic acid/3-CQA), providing a robust, Weight-of-Evidence (WoE) framework for the safety assessment of complex botanical matrices.

## 2. Materials and Methods

### 2.1. Preparation and Characterization of the Plant Extract

The species *B. trimera*, commonly known as “carqueja-amarga,” was obtained from standardized cultivation and registered at the Centro Pluridisciplinar de Pesquisas Químicas, Biológicas e Agrícolas (CPQBA–UNICAMP). The extract was prepared from aerial parts according to the percolation method described in the Brazilian Pharmacopoeia, 6th edition, volume 1 [24]. The plant material was ground using a knife mill (Tecnal, Piracicaba, Brazil), and a 20 g portion was macerated with 50% ethanol (Synth, São Paulo, Brazil) for approximately 24 h. Subsequently, extraction was performed in a stainless-steel percolator (Steel Service, São Paulo, Brazil) with additional solvent until 97 mL was collected by constant dripping. This first extract fraction was reserved, and percolation continued until the eluate became nearly colorless. The second fraction (approximately 1000 mL) was concentrated using a rotary evaporator (Heidolph, Schwabach, Germany) at 50 °C, 50 rpm, and approximately 100 mbar until a final volume of 83 mL was obtained. Both fractions were then combined and adjusted to a final volume of 200 mL to produce the extract used for subsequent characterization and testing.

Characterization of the extract included the determination of dry residue, density, and pH, as described in the Brazilian Pharmacopoeia [24], as well as exploratory liquid chromatography using variable gradient conditions and a C18 column (250 mm × 6 mm, 5 µm; ACE^®^, Advanced Chromatography Technologies, Aberdeen, UK). All analyses were performed using an Agilent Infinity 1260 chromatograph equipped with an autosampler, quaternary pump, column oven, and diode-array detector (Palo Alto, CA, USA), operated with OpenLab CDS software, version 2.2 (Agilent Technologies, Palo Alto, CA, USA). Chromatographic conditions were maintained at a flow rate of 0.8 mL/min, an injection volume of 20 µL, and constant inlet and outlet temperatures of 30 °C. Quantification of chlorogenic acid was conducted using the same system and calibration curves prepared from standard solutions at different concentrations, with emphasis on the levels present in the plant material.

The analysis of the *B. trimera* extract focused on chlorogenic acid and its isomers. Molecular structures were retrieved from the PubChem database, and the commercially purchased reference standard was identified as 3-caffeoylquinic acid (3-CQA), which served as the primary chemical marker for assessing the sensitization potential of the extract.

### 2.2. DPRA Proficiency and Extract Testing

To demonstrate assay proficiency and full compliance with OECD testing guidelines, a specific chemical panel comprising three proficiency substances and one positive control was utilized (Table 1). The proficiency substances included 1-chloro-2,4-dinitrobenzene (97%), classified as an extreme skin sensitizer, and butane-2,3-dione (97%), classified as a weak skin sensitizer, both purchased from Sigma-Aldrich (Cotia, Brazil), alongside butan-1-ol (99.5%), classified as a non-sensitizer, obtained from Merck (Rio de Janeiro, Brazil). Cinnamaldehyde (99%), also from Sigma-Aldrich, was employed strictly as the assay’s positive control. The synthetic nucleophilic targets used to evaluate protein-binding capacity, lysine and cysteine heptapeptides (Ac-RFAAKAA-COOH, >90% purity, and Ac-RFAACAA-COOH, >90% purity, respectively), were supplied by Biomatik (Wilmington, DE, USA). The remaining chemicals used in this study were analytical-grade reagents, buffers, or solvents required for sample preparation and mobile phase composition, which included: trifluoroacetic acid and ammonium hydroxide (28–30%) from Sigma-Aldrich; monosodium phosphate monohydrate, disodium phosphate heptahydrate, and acetonitrile (UV/HPLC grade) from Dinâmica (Campinas, Brazil); and ammonium acetate from Synth (Diadema, Brazil).

Chromatographic analyses were performed using the same equipment described for the characterization of the plant extract. A C18(2) column (3 × 150 mm, 5 µm; Phenomenex Luna^®^, Torrance, CA, USA) was employed, with the column oven maintained at 30 °C and detection carried out at 220 and 258 nm. The injection volume was adjusted from 7 µL to 10 µL, and the flow rate from 0.35 mL/min to 0.8 mL/min, due to differences between the available chromatograph and column and those originally described. Mobile phase A consisted of 0.1% (*v*/*v*) trifluoroacetic acid in water, whereas mobile phase B contained 0.085% (*v*/*v*) trifluoroacetic acid in acetonitrile. The gradient program was as follows: 0–10 min (10–25% B); 10–11 min (25–90% B); 11–13 min (isocratic); 13–13.5 min (90–10% B); and 13.5–20 min (isocratic). Chromatographic system suitability was assessed according to the acceptance criteria established in OECD TG 442C [5] and the EURL ECVAM DB-ALM Protocol n° 154 [25], including calibration linearity (r^2^ > 0.99), mean peptide concentration of Reference Controls A and C (0.50 ± 0.05 mM), and coefficient of variation (CV) of peptide peak areas for Reference Controls B and C in acetonitrile (CV < 15%). These parameters collectively ensure the specificity, precision, and quantitative accuracy of the analytical system for peptide depletion measurement. Full analytical validation of the DPRA chromatographic method, including LOD, LOQ, and robustness, has been previously established during the EURL ECVAM validation study and is documented in the referenced guideline.

Stock solutions of cysteine and lysine (0.667 mM) were prepared in sodium phosphate buffer (100 mM, pH 7.5) and ammonium acetate buffer (100 mM, pH 10.2), respectively. Test chemicals, including cinnamaldehyde as the positive control, were individually dissolved in 3 mL of acetonitrile at a concentration of 100 mM. For the cysteine peptide assay, samples were prepared by mixing 750 µL of the stock solution, 200 µL of acetonitrile, and 50 µL of the test chemical. For the lysine peptide assay, 750 µL of the stock solution was combined with 250 µL of the test chemical.

Calibration curves for the peptides were prepared by serial dilution of the stock solutions, as described in OECD TG 442C [5]. Reference controls were used to assess changes in peptide stability unrelated to the added chemicals. Control A, designed to ensure calibration curve accuracy, was prepared by combining 750 µL of the peptide stock solution with 250 µL of acetonitrile. Control B was prepared identically to Control A but injected at the beginning and end of each run to monitor peptide stability. Control C was prepared separately for each solvent used to dissolve the test chemicals (in the proficiency assay, acetonitrile only), replacing acetonitrile in the mixture to verify that the solvent did not affect peptide depletion; its analysis was performed alongside the samples.

Co-elution controls were prepared by replacing the peptide stock solutions with the corresponding buffers to determine the chromatographic profile of each test chemical and to assess whether its retention time overlapped with that of the peptides. Both reference control samples and test chemical samples were prepared in triplicate, except for Control B, which was analyzed in six replicates, comprising one triplicate at the beginning and another at the end of each run to assess peptide stability throughout the analytical run. After sample preparation, the solutions were incubated in a water bath at 25 °C for 24 h and subsequently analyzed by HPLC.

Peptide depletion was calculated for each sample replicate according to OECD TG 442C [5], using the mean peak area of Control C as reference. All measurements were performed in triplicate (n = 3), unless otherwise stated. The mean, standard deviation (SD), and CV were calculated for each set of replicates. The CV was used to assess the repeatability of the measurements against the acceptance criteria established by OECD TG 442C [5]. The CV of Control B peak areas were calculated separately for each triplicate set.

Following DPRA validation, the assay was applied to assess the response of Key Event 1 of the skin sensitization AOP and to evaluate the dermal sensitization potential of the obtained extract and its major component [26]. The extract used in this assay consisted of a hydroethanolic extract (HAE) of *B. trimera* aerial parts, prepared by percolation with 50% ethanol according to the Brazilian Pharmacopoeia, 6th edition, as described in Section 2.1. For this purpose, the standardized extract was prepared in 3 mL of 50% ethanol at a concentration of 5%, which corresponds to the maximum level commonly used for plant extracts in cosmetic formulations. Based on the quantification of chlorogenic acid in the *B. trimera* extract, the amount of this compound present in the 5% extract was calculated, and a chlorogenic acid standard solution was prepared in 50% ethanol at the corresponding concentration.

To evaluate both samples, a second HPLC run was performed following the same analytical sequence established for the proficiency chemicals, while maintaining the calibration curve and run controls. Accordingly, two co-elution controls consisted of the extract and chlorogenic acid samples without peptide addition, and an additional Control C containing 50% ethanol was included, alongside the acetonitrile control used to solubilize the positive control.

### 2.3. In Silico Analysis

Computational predictions were executed by inputting the Simplified Molecular-Input Line-Entry System (SMILES) codes or Chemical Abstracts Service (CAS) registry numbers compiled in Table 1. Molecular structures and SMILES codes were retrieved from the PubChem database, while CAS numbers were verified against the European Commission’s CosIng database. To guarantee the regulatory validity and reliability of the outputs, data acceptance was strictly conditioned upon the model’s applicability domain (AD) and a predefined confidence threshold. The AD was evaluated for each individual platform to ensure that the target benchmark substances and plant phytochemicals fell within the structural, interpolation, and chemical descriptor spaces of the underlying training sets, thereby preventing mathematical extrapolation errors. Consequently, only predictions verified to be structurally within the AD and presenting a confidence level of preferably equal to or greater than 70% (≥70%) were considered for the predictive matrix. This specific threshold aligns with international regulatory screening recommendations, which accept a confidence level ≥ 70% as a robust, high-stringency baseline for WoE decisions within cosmetic safety assessment frameworks, successfully balancing predictive sensitivity with screening efficiency [23,27,28]. To construct a robust computational tier for the proposed ITS, this computational panel was designed to operate under an integrated multi-tool framework, providing consensus data aligned with the Defined Approaches (DA) recommendations under OECD Test Guideline 497 [27].

The in silico evaluation of all compounds presented in Table 1 was conducted using six software tools for skin sensitization hazard assessment, collectively acting as the predictive core of the ITS framework: ADMET Predictor™ (Simulations Plus Inc., Research Triangle Park, NC, USA, version 9.5), OECD QSAR Toolbox (OECD, version 4.8.2; https://qsartoolbox.org/, accessed on 4 May 2024), StopTox (https://stoptox.mml.unc.edu/, accessed on 12 July 2025), Pred-Skin (LABMOL, version 3.0 https://predskin.labmol.com.br/, accessed on 14 June 2025), Vega QSAR (version 1.2.0; https://www.vegahub.eu/portfolio-item/vega-qsar/, accessed on 4 May 2023), and Danish QSAR (https://qsardb.food.dtu.dk/db/index.html, accessed on 4 May 2023). The computational panel employed comprises both open-source and commercial platforms. Tools such as the OECD QSAR Toolbox, Vega QSAR, and Danish QSAR are open-access, providing high transparency and comprehensive QMRF (QSAR Model Reporting Format) documentation, which facilitates regulatory acceptance. Conversely, ADMET Predictor™ is a commercial tool; while it operates as a ‘black box’ regarding proprietary algorithms, its predictive power relies on validated, high-quality datasets and rigorous internal documentation, consistent with its extensive use in industry safety pipelines. StopTox and Pred-Skin represent intermediate approaches, offering clear methodological documentation regarding their descriptor-based mechanisms. This mix ensures a balanced evaluation: leveraging the open transparency of OECD-supported tools with the high-performance capabilities of commercial software.

In ADMET Predictor™, after uploading the molecular structure files, physiological pH (7.4) was selected, and data on lipophilicity (logP), skin permeation, and skin sensitization were obtained. Within the OECD QSAR Toolbox, predictions for the skin sensitization endpoint were executed via an automated workflow specifically designed for regulatory integrated frameworks: Data gap filling method: Read-across analysis, Automated workflow for EC3 from LLNA or Skin sensitization from GPMT assays for defined approaches (SS AW for DASS). Conversely, for the remaining in silico software platforms, all available predictive models were fully considered to ensure a comprehensive screening consensus within the WoE matrix.

## 3. Results and Discussion

### 3.1. Partial DPRA Proficiency

**Figure 1:**
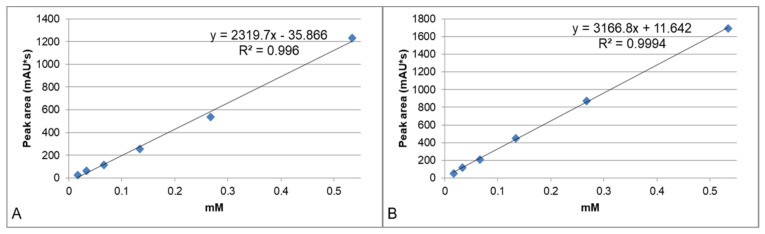
Calibration curves for cysteine (**A**) and lysine (**B**) peptides.

For both peptides, the analytical response exhibited linear behavior, with coefficients of determination R^2^ > 0.99, indicating that the chromatographic peak area was proportional to peptide concentration. This linearity enabled reliable quantification of the peptides in the analyzed solutions, as illustrated in Figure 1.

None 0. ± 0.05 mM) and the coefficient of variation of peak areas in controls B and C (<15%), as presented in Table 2.

**Figure 2:**
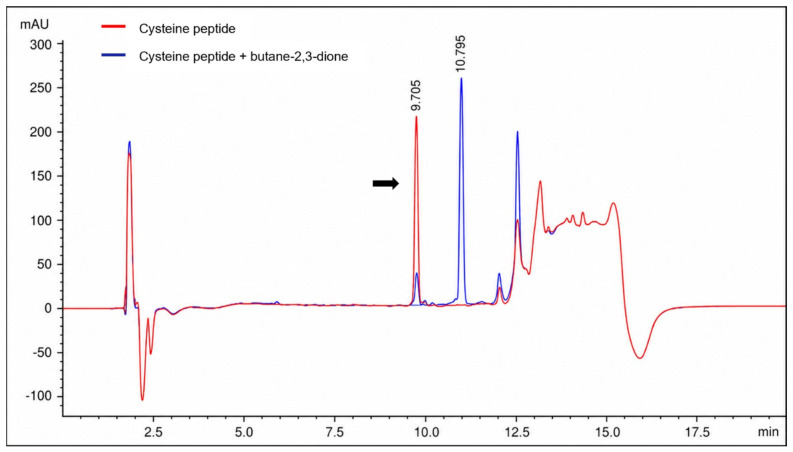
Chromatographic profile (220 nm) shows cysteine peptide depletion after reaction with butane-2,3-dione at 100 mM. The arrow indicates peptide depletion. The red peak corresponds to the native peptide, whereas the blue peak represents the peptide concentration following exposure to the depleting agent.

The performance of the assay with the proficiency chemicals was satisfactory (as illustrated in Figure 2), meeting the acceptance criteria for the standard deviation of cysteine and lysine depletion values (<14.9% and <11.6%, respectively) in all cases (Table 3). The calculated depletion values were consistent with those recommended by the guideline, except for the lysine peptide reacted with cinnamaldehyde (positive control). During the preparation of these samples, slight turbidity was observed, unlike in the cysteine assay, which may be associated with the lower-than-expected depletion (18.31% compared with 40.2%).

According to OECD TG 442C [5], depletion values are used to classify test substances into one of four reactivity categories that discriminate sensitizers from non-sensitizers. Reactivity classification was performed using the Cysteine 1:10/Lysine 1:50 prediction model, in which the mean of the percent cysteine and percent lysine peptide depletion values is calculated for each test substance, with negative depletion values considered as zero. The resulting mean depletion value is assigned to one of four reactivity categories: minimal (<6.38%), low (6.38% ≤ depletion ≤ 22.62%), moderate (22.62% < depletion ≤ 42.47%), or high (depletion > 42.47%). Substances assigned to the minimal reactivity category are classified as non-sensitizers, whereas those assigned to low, moderate, or high reactivity categories are classified as sensitizers. This model confirmed the experimental results obtained: butan-1-ol was classified as minimal reactivity (non-sensitizer), whereas cinnamaldehyde, butane-2,3-dione, and 1-chloro-2,4-dinitrobenzene were classified as high reactivity (sensitizers).

The in chemico proficiency assay successfully predicted the sensitization potential of all four tested substances, confirming the reliability of our experimental setup. As expected, the reference sensitizers 1-chloro-2,4-dinitrobenzene and cinnamaldehyde exhibited high reactivity, evidenced by significant peptide depletion and, in the case of 1-chloro-2,4-dinitrobenzene, the formation of precipitates in the cysteine reaction vial. Butane-2,3-dione, classified as a weak/moderate sensitizer, showed a lower but distinct reactivity profile. In contrast, the non-sensitizer butan-1-ol demonstrated negligible peptide depletion, falling well within the established threshold for non-sensitizing substances.

A nuanced observation emerged when comparing the experimental DPRA depletion of 1-chloro-2,4-dinitrobenzene with the calculated in silico sensitization probability of butane-2,3-dione (Table 4). While 1-chloro-2,4-dinitrobenzene exhibited substantially higher experimental chemical reactivity, butane-2,3-dione displayed a higher theoretical sensitization probability. This discrepancy highlights that sensitization risk is not solely driven by peptide reactivity; as shown in Table 4, butane-2,3-dione presents a superior dermal permeation value. This enhanced permeation capacity is not derived from its lipophilicity, as its logP is lower than that of 1-chloro-2,4-dinitrobenzene, but is primarily a consequence of its lower molecular weight. These findings align with the principles established by Roberts and Aptula [29], reinforcing that skin penetration is a multifactorial process governed by both molecular size and hydrophobicity, and that experimental in chemico reactivity must be interpreted alongside physical transport parameters to accurately estimate sensitizing potency.

### 3.2. Assessment of Sensitization Potential Using In Silico Tools

In line with modern regulatory paradigms, the SCCS Notes of Guidance for the Testing of Cosmetic Ingredients and Their Safety Evaluation (12th Revision) [28] explicitly highlights that a single in silico model is rarely sufficient to provide a definitive toxicological conclusion. Therefore, an ITS using a WoE framework is highly recommended [23,28]. Our study adhered to this principle by employing a battery of six complementary computational platforms: ADMET Predictor™, OECD QSAR Toolbox, StopTox, Pred-Skin, Vega QSAR, and Danish QSAR. This approach is strategically coupled with an expert rule-based system (OECD QSAR Toolbox), which identifies localized structural alerts for protein binding, with statistical and machine-learning models that evaluate global molecular descriptors and probabilistic historical data. To address the variability across models, we adopted a consensus-based approach. In cases of discordant predictions, we prioritized the results from tools with higher structural similarity to the target compounds, as defined by their specific applicability domains. This allows us to determine not just ‘what’ the model predicts, but ‘why’ specific chemical features (e.g., electrophilic centers) are triggering those predictions, thereby improving predictive reliability.

Evaluating skin sensitization through multiple computational platforms inherently introduces challenges regarding model uncertainties and conflicting predictions, which are evident in our dataset (Table 4). For instance, the reference sensitizers 1-chloro-2,4-dinitrobenzene (DNCB) and cinnamaldehyde were unanimously and correctly predicted as positive by all applicable platforms, often with high confidence levels or categorized as high-risk (Category 1A). Similarly, the non-sensitizer butan-1-ol was consistently predicted as negative across all tools. However, conflicting predictions emerged for butane-2,3-dione, a weak/moderate sensitizer: while ADMET Predictor, QSAR Toolbox, Vega QSAR, and Danish QSAR correctly predicted a positive outcome, StopTox classified it as negative, and it fell outside the applicability domain of Pred-Skin. Such discrepancies underline the necessity of the WoE framework, where a single ‘consensus’ vote is replaced by professional judgment that weighs the transparency of each model, its specific applicability domain, and the biological relevance of the detected structural features.

Beyond binary classification in silico tools provided crucial mechanistic and physicochemical data to elucidate the observed in chemico reactivity. The OECD QSAR Toolbox and Pred-Skin successfully identified specific protein-binding alerts and GHS classifications, respectively, which align with the United Nations Globally Harmonized System (GHS) risk framework. Within this regulatory context, Risk Category 1A comprises substances with high sensitizing potential, whereas Risk Category 1B comprises substances with moderate to low sensitization potential. Specifically, the OECD QSAR Toolbox identified that 1-chloro-2,4-dinitrobenzene (DNCB)—a doubly substituted aromatic ring bearing a strongly electron-withdrawing nitro group—exhibits high reactivity toward nucleophilic aromatic substitution (NAS) reactions, correlating with its subcategorization as a high-potency Category 1A sensitizer. Conversely, butane-2,3-dione was identified via the QSAR Toolbox as acting through a Schiff base formation mechanism—a pathway characteristic of the lower-potency Category 1B classification—while butan-1-ol presented no structural alerts or risk designations due to its non-reactive nature.

Furthermore, physicochemical descriptors generated by ADMET Predictor™ (S+logP and skin permeation) highlighted that skin penetration is primarily influenced by hydrophobicity and molecular weight [29]. Interestingly, while 1-chloro-2,4-dinitrobenzene (DNCB) exhibited substantially higher experimental reactivity in the DPRA than butane-2,3-dione, the latter displayed a higher calculated skin permeation value, likely due to its lower molecular weight. As corroborated by Roberts and Aptula [29], these results show that there is no strict linear correlation between logP and sensitizing potency, as strong sensitizers often display either very high or very low logP values. Ultimately, the inherent ability of a substance to covalently bind to nucleophilic sites remains the key determinant for sensitization [26].

Regarding the phytochemical markers of the *B. trimera* extract (Table 4), the in silico predictions for chlorogenic acid (3-CQA) and its caffeoylquinic derivatives (3,5-diCQA, 4,5-diCQA, and 3,4,5-triCQA) highlighted the inherent challenges of modeling complex polyphenolic structures. For the isolated 3-CQA, ADMET Predictor™ was the only computational tool to accurately forecast the negative sensitization potential (73% confidence), aligning perfectly with the experimental in chemico outcome. Conversely, the other platforms either predicted a false-positive outcome (predominantly categorizing it as a weak Category 1B sensitizer) or identified the molecule as outside their applicability domain (e.g., OECD QSAR Toolbox). Interestingly, as structural complexity increased with the addition of caffeoyl groups in the derivatives, a notable predictive shift occurred: most platforms (ADMET Predictor, QSAR Toolbox, StopTox, Pred-Skin, and Danish QSAR) reached a consensus, flagging the di- and tri-caffeoylquinic acids as positive sensitizers. This shift is strongly supported by the physicochemical descriptors generated by ADMET Predictor, which revealed a clear trend: the sequential addition of caffeoyl moieties drastically increased both lipophilicity (S+logP rising from −0.398 in 3-CQA to 2.968 in 3,4,5-triCQA) and theoretical skin permeation (from 0.153 to 264.818 cm/s × 10^−7^). This enhanced theoretical capacity to penetrate the stratum corneum likely drives the predictive models to classify these larger, more lipophilic derivatives as potential skin sensitizers, demonstrating how in silico descriptors can guide the toxicological profiling of complex botanical mixtures.

While in silico tools provide robust mechanistic insights, neither computational predictions nor DPRA experimental results can be used in isolation to definitively subcategorize substances or predict sensitization potency for final safety assessments [27]. However, following the principles of OECD Test Guideline 497 [27], these tools can be successfully incorporated into a defined ITS. By establishing a rational methodological sequence that combines the identification of mechanistic domains, structural alerts, and experimental protein-binding data (Key Event 1), this integrated approach provides a reliable, animal-free screening matrix. This framework not only fulfills regulatory requirements for hazard identification but also enables mechanistic read-across to predict the sensitization potential of complex or untested cosmetic ingredients.

### 3.3. Toxicological Profiling and Integrated Analysis of B. trimera Extract, Chlorogenic Acid, and Its Isomers

Among the key events in the skin sensitization pathway, the second involves the activation of the detoxification response in keratinocytes by electrophilic compounds through the modification of the Keap1/Nrf2 complex [4]. This transcription factor regulates the antioxidant response element (ARE) and promotes the transcription of phase II detoxification enzymes [30]. This pathway is typically assessed using OECD Test Guideline 442D [30], which employs genetically modified keratinocytes that emit bioluminescence upon pathway activation. Although polyphenols are widely recognized for their antioxidant and anti-inflammatory properties, these compounds meet the requirements for pathway activation. This occurs because they are electrophilic, exhibit metal-chelating activity, and react with thiol groups, ultimately leading to protein haptenation [21].

Experimentally, the *B. trimera* hydroethanolic extract (HAE) presented a dry residue of 0.0192 ± 0.02534 g/g and a density of 1.0116 g/mL at 26.5 °C, with a total chlorogenic acid content of 0.27% and an acidic pH of 4.59, which likely reflects the presence of constituent phenolic acids. In the DPRA, the HAE was classified as a sensitizer, albeit displaying a low-potency peptide depletion value of 11.5%. Interestingly, the major constituent, chlorogenic acid (3-CQA), yielded a completely negative result. This apparent discrepancy demonstrates the inherent limitation of relying solely on isolated chemical markers to characterize crude extracts.

To resolve this limitation, the integration of in chemico and in silico approaches within an ITS becomes essential. While the DPRA provides direct, indispensable experimental evidence regarding the protein reactivity of the primary accessible standard (3-CQA), the multi-tool in silico battery allows us to expand the toxicological screening to those chlorogenic acid isomers that are not commercially available for bench testing. This dual-layered framework—coupling physical testing of the major marker with broad computational profiling of the secondary constituents—substantially minimizes the risk of false negatives, thereby increasing the overall accuracy and regulatory confidence in the hazard evaluation of the complex botanical matrix.

The in silico profiling of 3-CQA and its caffeoylquinic derivatives (3,5-diCQA, 4,5-diCQA, and 3,4,5-triCQA) provided critical mechanistic insights into the driving factors behind the HAE sensitization potential (Table 4). For the major constituent (3-CQA), ADMET Predictor™ accurately forecasted a negative sensitization outcome with 73% confidence, which was validated by the experimental DPRA depletion value of −1.26% (minimal reactivity). Although the OECD QSAR Toolbox identified a theoretical structural alert for Michael addition (Category 1B) for 3-CQA, this potential reactivity did not translate into physical peptide depletion.

Conversely, as structural complexity increased across the derivatives, a distinct predictive trend emerged: the majority of the computational platforms reached a consensus, flagging di- and tri-caffeoylquinic acids as potential sensitizers. This shift is strongly supported by the ADMET Predictor™ physicochemical descriptors, which revealed that the sequential addition of caffeoyl moieties significantly increased both lipophilicity (S+logP rising from −0.398 in 3-CQA to 2.968 in 3,4,5-triCQA) and estimated skin permeation (increasing from 0.153 × 10^−7^ cm/s to 264.818 × 10^−7^ cm/s). These findings suggest that while the primary marker is non-sensitizing, the heightened lipophilicity and molecular weight of the co-existing derivatives likely facilitate their penetration through the stratum corneum and subsequent interaction with dermal protein nucleophiles. Consequently, the positive sensitization result of the crude HAE is not governed by 3-CQA alone, but rather by the cumulative and synergistic contributions of these minor, more hydrophobic isomers [31].

Beyond enhanced skin permeation, the structural evolution from mono- to poly-caffeoyl derivatives introduces a critical chemical variable related to the pro-hapten activation mechanism of catechols. Phenolic structures bearing catechol rings are not inherently highly electrophilic; rather, they require chemical or enzymatic oxidation to transform into reactive ortho-quinones, which act as potent Michael acceptors capable of protein haptenation [21,29]. In this context, the presence of multiple caffeoyl moieties in di-CQA and tri-CQA structurally multiplies the reactive centers per molecule compared to 3-CQA. This multiplication significantly accelerates cumulative auto-oxidation rates and enables polyvalent cross-linking with protein nucleophiles [29].

Furthermore, this mechanism highlights the role of experimental boundary conditions in assay sensitivity. While the DPRA lysine assay operates at an elevated pH of 10.2 to promote nucleophilic reaction with amines, the cysteine assay is restricted to pH 7.5 [5,25]. At this physiological pH, the auto-oxidation kinetics of a single catechol ring (3-CQA) may be too slow to trigger significant peptide depletion within the standard 24 h incubation, leading to the observed negative in chemico outcome. Conversely, in silico platforms evaluate hazard based on structural alerts for pro-hapten bioactivation pathways (i.e., Michael addition via catechol transformation) independent of experimental pH limitations [26]. Subtle structural variations among the isomers and local pH environments can thus generate highly variable peptide depletion profiles, a phenomenon that underscores why relying solely on a single isolated marker under rigid assay conditions can mask the true sensitization risk of a multi-component botanical extract.

The robustness of this six-tool in silico panel was further confirmed through the proficiency assay (Table 4), which showed high predictive consensus for the control substances, validating the overall reliability of the proposed ITS [23,27]. Reference sensitizers, such as 1-chloro-2,4-dinitrobenzene and cinnamaldehyde, were correctly classified as Category 1A sensitizers, while butan-1-ol was consistently identified as a non-sensitizer. Because the computational panel demonstrated high accuracy in distinguishing chemical reactivity profiles within simple matrices, the predicted divergence between 3-CQA and its di- and tri-caffeoylquinic derivatives gains significant scientific weight. This successful validation ensures that the sensitization predictions for the larger derivatives are not mathematical artifacts, but rather a faithful reflection of shifts in physicochemical and reactive properties, fully aligned with the AOP framework for skin sensitization [26].

## 4. Conclusions

This study successfully implemented and validated an Integrated Testing Strategy (ITS) for skin sensitization screening, combining in chemico (DPRA) and in silico approaches. The demonstration of partial DPRA proficiency and the high predictive consensus across the six-tool in silico panel confirm the reliability of the integrated framework for hazard identification. Notably, the integration of computational tools proved essential for interpreting experimental outcomes in complex botanical matrices, where the sensitization potential of the *B. trimera* extract could not be attributed to a single marker (3-CQA) but rather to the cumulative reactivity and enhanced skin permeation of its caffeoylquinic derivatives.

These findings underscore the necessity of a multi-tool Weight-of-Evidence (WoE) framework over reliance on isolated markers or single predictive models. While not intended as a replacement for definitive clinical or higher-tier assays, this ITS offers a time- and cost-efficient screening strategy for the early exclusion of unsuitable cosmetic ingredients. Furthermore, our results highlight the need for a critical reassessment of the applicability domains of existing validated assays when applied to plant-derived materials, particularly regarding how rigid experimental boundary conditions, such as assay pH, can limit the in chemico detection of pro-hapten auto-oxidation kinetics. By bridging the gap between isolated marker testing and the complex reactive landscape of botanical mixtures, this study provides a robust, animal-free methodological path for the safety evaluation of cosmetic raw materials.

Finally, it is important to acknowledge that the present study demonstrated DPRA proficiency using three of the ten recommended reference substances. While these results provide an initial validation of the experimental setup, further research is required to expand this demonstration to the complete set of reference substances. Future studies should aim to broaden the chemical diversity of the proficiency panel to further enhance the reliability and robustness of this integrated approach, particularly when applied to the complex matrices inherent to plant-derived extracts such as *B. trimera*. In this context, the present ITS is intended as a foundational framework; future investigations involving larger datasets of botanical constituents will be the logical next step to further broaden the applicability domain and perform extensive external benchmarking, reinforcing regulatory confidence in these animal-free methodologies.

## Figures and Tables

**Table 1:** Chemical information of proficiency substances, 3-Caffeoylquinic acid (chlorogenic acid), and its isomers.

Substance	PubChem CID	SMILES Molecule	CAS Number
1-chloro-2,4-dinitrobenzene	6	C1=CC(=C(C=C1[N+](=O)[O-])[N+](=O)[O-])Cl	97-00-7
Cinnamaldehyde	637511	C1=CC=C(C=C1)/C=C/C=O	104-55-2
Butane-2,3-dione	650	CC(=O)C(=O)C	431-03-8
Butan-1-ol	263	CCCCO	71-36-3
3-Caffeoylquinic acid (Chlorogenic acid/3-CQA)	1794427	C1[C@H]([C@H]([C@@H](C[C@@]1(C(=O)O)O)OC(=O)/C=C/C2=CC(=C(C=C2)O)O)O)O	327-97-9
3,5-di-O-caffeoylquinic acid (3,5-diCQA)	6474310	C1C(C[C@H](C([C@@H]1OC(=O)/C=C/C2=CC(=C(C=C2)O)O)O)OC(=O)/C=C/C3=CC(=C(C=C3)O)O)(O)C(=O)O	24512-63-8
4,5-di-O-caffeoylquinic acid (4,5-diCQA)	5281780	C1[C@H]([C@H]([C@@H](C[C@@]1(C(=O)O)O)OC(=O)/C=C/C2=CC(=C(C=C2)O)O)OC(=O)/C=C/C3=CC(=C(C=C3)O)O)O	57378-72-0
3,4,5-tri-O-caffeoylquinic acid(3,4,5-triCQA)	6440783	C1C(C[C@H](C([C@@H]1OC(=O)/C=C/C2=CC(=C(C=C2)O)O)OC(=O)/C=C/C3=CC(=C(C=C3)O)O)OC(=O)/C=C/C4=CC(=C(C=C4)O)O)(O)C(=O)O	86632-03-3

**Table 2:** Values of reference controls A, B, and C for both peptides in the DPRA validation. SD and CV were calculated from the percentage of peptide depletion values obtained for each sample replicate (n = 3).

	Cysteine			Lysine		
	A	B	C	A	B	C
**Mean peak area**	1166.31	1083.66	1132.00	1622.33	1544.26	1617.42
**Mean peptide concentration (mM)**	0.51	0.48	0.5	0.5	0.48	0.5
**Standard deviation (SD)**	21.00	86.02	23.02	3.85	168.21	41.65
**Coefficient of variation (%) (CV)**	1.8	7.94	2.03	0.24	10.89	2.58

**Table 3:** Depletion values for the positive control (cinnamaldehyde) and proficiency chemicals.

	Cinnamaldehyde		Butane-2,3-Dione		1-Chloro-2,4-Dinitrobenzene (DNCB)		Butan-1-ol	
	Cysteine	Lysine	Cysteine	Lysine	Cysteine	Lysine	Cysteine	Lysine
**Mean depletion (%)**	77.64 ^a^	18.31 ^b^	85.50 ^c^	10.95 ^d^	99.02 ^e^	14.93 ^f^	8.20 ^g^	0.26 ^h^
**DP**	1.27	3.99	3.84	0.16	0.46	1.52	0.10	1.58
**CV (%)**	1.64	21.79	4.49	1.46	0.46	10.181	1.17	615.44
**Mean depletion**	47.98		48.23		56.97		4.23	

DP: Standard deviation. CV: Coefficient of variation. Mean depletion (%), SD, and CV were calculated from the percentage of peptide depletion values obtained across the three replicates of each sample (n = 3). Expected value ranges established by OECD TG 442C for each substance: ^a^ 60.8–100.0. ^b^ 40.2–69.4. ^c^ 60–100. ^d^ 10–45. ^e^ 90–100. ^f^ 15–45. ^g^ ≤7. ^h^ ≤5.5.

**Table 4:** Comparison of in silico predictions and experimental in chemico reactivity data for proficiency substances and *B. trimera* constituents.

	In Silico Tools									In Chemico Test
	ADMET Predictor™				QSAR Toolbox	StopTox	Vega QSAR	Pred-Skin	Danish QSAR	DPRA
	Molecular Weight (g/mol)	Skin Sensitization (Probability)	Skin Permeation (cm/s × 10^−7^)	S+logP	Protein-Binding Alert	Skin Sensitization (Confidence)	Skin Sensitization (Confidence)	Skin Sensitization (Confidence)	Skin Sensitization	Sensitization Potential (Depletion/Reactivity)
1-chloro-2,4-dinitrobenzene	202.55	Positive −88%	16.228	2.15	Positive	Positive −100%	Positive (AD Index: 1)	Positive-Cat 1A (87.3%)	Positive	Positive (56.97%/High)
Butane-2,3-dione	86.09	Positive −99%	63.512	−497	Positive	Negative −80%	Positive (AD Index: 1)	Out of domain	Positive	Positive (48.23%/High)
Butan-1-ol	74.12	Negative (97%)	1.597	733	Negative	Negative −90%	Negative (AD Index: 1)	Negative −90.80%	Negative	Negative (4.23%/Minimum)
Cinnamaldehyde	132,16	Positive −99%	15.861	1.964	Positive	Positive (100%)	Positive (AD Index: 1)	Positive-Cat 1A (81.3%)	Positive	Positive (47.98%/High)
3-CQA	354.34	Negative −73%	0.153	−0.398	Out of domain	Positive −60%	Positive (AD Index: 0.86)	Positive-Cat 1B (69.4%)	Positive	Negative (−1.26%/Minimum)
3,5-diCQA	516.46	Positive (N.D.)	25.026	1.167	Positive	Positive −60%	Out of domain	Positive-Cat 1B (70.1%)	Positive	Not tested
4,5-diCQA	516.46	Positive (N.D.)	25.856	1.151	Positive	Positive −60%	Out of domain	Positive-Cat 1B (67.3%)	Positive	Not tested
3,4,5-triCQA	578.6	Posit (N.D.)	264.818	2.968	Positive	Positive −70%	Out of domain	Positive-Cat 1B (70.7%)	Positive	Not tested
*B. trimera* HAE	Not tested	Not tested	Not tested	Not tested	Not tested	Not tested	Not tested	Not tested	Not tested	Positive (11.51%/Low)

HAE: hydroalcoholic extract; S+logP: ADMET Predictor™ predicted octanol–water partition coefficient, representing the molecular lipophilicity index; AD Index: The AD Index (Applicability Domain Index) represents the reliability of the prediction based on the structural similarity of the query compound to the training set of the model. An index close to 1 indicates high structural similarity and, consequently, a more reliable prediction, whereas values close to 0 suggest low similarity and higher uncertainty; N.D.: not determined.

## Data Availability

The original contributions presented in this study are included in the article. Further inquiries can be directed to the corresponding author.

## Abbreviations

LLNA	Local Lymph Node Assay
AOP	Adverse Outcome Pathway
DPRA	Direct Peptide Reactivity Assay
NAMs	New Approach Methodologies
OECD	Organisation for Economic Co-operation and Development
HPLC	High-performance Liquid Chromatography
IATA	Integrated Approaches to Testing and Assessment
SMILES	Simplified Molecular-Input Line-Entry System
AD	Applicability domain
DP	Standard Deviation
CV	Coefficient of variation
MoS	Margin of safety
TTC	Threshold of toxicological concern
NAS	Nucleophilic aromatic substitution
ITS	Integrated Testing Strategy
ARE	Antioxidant response element
